# GCC2 as a New Early Diagnostic Biomarker for Non-Small Cell Lung Cancer

**DOI:** 10.3390/cancers13215482

**Published:** 2021-10-31

**Authors:** Hyesun Jeong, Byeong Hyeon Choi, JinA Park, Jik-Han Jung, Hyunku Shin, Ka-Won Kang, Yu Hua Quan, Jewon Yu, Ji-Ho Park, Yong Park, Yeonho Choi, Hyun Koo Kim, Sunghoi Hong

**Affiliations:** 1 School of Biosystems and Biomedical Sciences, Korea University, Seoul 02841, Korea; lullaby0810@gmail.com (H.J.); 2020020815@korea.ac.kr (J.P.); 2 BK21 FOUR R&E Center for Precision Public Health, Graduate School of Korea University, Seoul 02855, Korea; yeonhochoi@korea.ac.kr; 3 Korea Artificial Organ Center, Korea University, Seoul 02841, Korea; baby2music@gmail.com; 4 Department of Thoracic and Cardiovascular Surgery, Korea University Guro Hospital, College of Medicine, Korea University, Seoul 08308, Korea; hwa1983418@gmail.com; 5 Department of Bio and Brain Engineering, Korea Advanced Institute of Science and Technology (KAIST), Daejeon 34141, Korea; jjhan@kaist.ac.kr (J.-H.J.); jihopark@kaist.ac.kr (J.-H.P.); 6 Department of Bio-Convergence Engineering, Korea University, Seoul 02841, Korea; ramgee91@gmail.com; 7 Department of Internal Medicine, Division of Hematology-Oncology, Korea University College of Medicine, Seoul 02841, Korea; ggm1018@gmail.com (K.-W.K.); paark76@korea.ac.kr (Y.P.); 8 Department of Biomedical Sciences, College of Medicine, Korea University, Seoul 02841, Korea; 9 Exopert Corporation 4th Floor, Seoul 02580, Korea; jewon@exopert.com

**Keywords:** exosomes, non-small cell lung cancer, GCC2, biomarkers, early detection, liquid biopsy, cancer

## Abstract

**Simple Summary:**

Lung cancer, including non-small cell lung cancer, is the leading cause of cancer-related death worldwide. A better prognosis is associated with early diagnosis of lung cancer patients. Although annual screening guidelines for lung cancer are recommended, using various tools such as chest X-ray, low-dose computed tomography, and positron emission tomography, these screening procedures are expensive and difficult to repeat. They are also invasive and have a high risk of radiation exposure. Therefore, a low-risk, convenient diagnostic method using liquid biopsy and biomarkers is required for the early diagnosis of lung cancer. The newly proposed biomarker GCC2 was identified through proteomic analysis of exosomes secreted from lung cancer cell lines. GCC2 expression levels in peripheral blood of the patients showed high specificity and sensitivity in early lung cancer, demonstrating that our novel exosomal biomarker GCC2 can greatly contribute to improving the diagnosis of lung cancer patients, even though it has been tested in only a few pilot studies.

**Abstract:**

No specific markers have been identified to detect non-small cell lung cancer (NSCLC) cell-derived exosomes circulating in the blood. Here, we report a new biomarker that distinguishes between cancer and non-cancer cell-derived exosomes. Exosomes isolated from patient plasmas at various pathological stages of NSCLC, NSCLC cell lines, and human pulmonary alveolar epithelial cells isolated using size exclusion chromatography were characterized. The GRIP and coiled-coil domain-containing 2 (GCC2) protein, involved in endosome-to-Golgi transport, was identified by proteomics analysis of NSCLC cell line-derived exosomes. GCC2 protein levels in the exosomes derived from early-stage NSCLC patients were higher than those from healthy controls. Receiver operating characteristic curve analysis revealed the diagnostic sensitivity and specificity of exosomal GCC2 to be 90% and 75%, respectively. A high area under the curve, 0.844, confirmed that GCC2 levels could effectively distinguish between the exosomes. These results demonstrate GCC2 as a promising early diagnostic biomarker for NSCLC.

## 1. Introduction

Lung cancer is a leading cause of cancer-related death in the world, with non-small cell lung cancer (NSCLC) accounting for almost 85% of all lung cancers [1,2]. NSCLC is further classified into adeno-, squamous cell, and large-cell carcinomas. Adenocarcinoma is the most common type of lung cancer, accounting for approximately 40% of all lung cancers (squamous cell carcinoma 25–30% and large cell carcinoma 5–10% of all lung cancers) [3]. Diagnosis at an early stage is associated with better prognosis in lung cancer patients [4]. Furthermore, markedly improved survival rates have been reported in patients with early-stage NSCLC after undergoing surgical resection [5,6,7].

Clinical guidelines recommend annual screening for lung cancer through multiple methods such as chest X-rays, low-dose computed tomography (LDCT) scan, positron emission tomography (PET-CT), magnetic resonance imaging (MRI), and bronchoscopic or CT-guided needle biopsies [8]. However, these examination procedures are expensive, difficult to repeat, invasive, and have a high risk of radiation exposure [9]. Therefore, low-risk and convenient diagnostic methods are required for early lung cancer diagnosis.

Liquid biopsies capture cancer-related biomarkers circulating in the body fluids such as exosomes, circulating tumor cells (CTCs), and circulating tumor DNA (ctDNA); they have great potential as an alternative diagnostic tool for various cancer types [10,11]. CTCs and ctDNAs are difficult to detect because of their extremely low concentrations in the blood, whereas exosomes are easy to detect because they are present in high concentrations, making them advantageous in liquid biopsies [12]. However, diagnosing NSCLC can still be challenging; therefore, new technology is required to improve the diagnostic work-up and eventually enhance targeted therapies for cancer.

Cells secrete extracellular vesicles, such as nano-sized exosomes with diameters of 30–150 nm [13,14], containing nucleic acids and proteins [15]. Exosomes are released into the extracellular space and then enter the blood to be circulated [16]. Studies have shown that exosomes act as modulators of cell-to-cell communication and intracellular biological activity [17,18]. Exosomes have cup-shaped structures when observed under a transmission electron microscope. These heterogeneous membrane-enclosed vesicles contain exosome-enriched proteins including the tetraspanin family (CD63, CD9, and CD81), heat shock proteins (HSP60, HSP70, and HSP90), and members of endosomal sorting complexes, such as TSG101 and Alix, required for transport [19]. Moreover, exosomes can be easily isolated from blood samples of cancer patients, making them an effective biomarker [20,21,22,23]. GCC2 is a Golgi-localized protein that has been shown to form a long coiled-coil structure that protrudes from the Golgi surface [24], while also being involved in Golgi structure maintenance [25,26]. Interestingly, a recent study has identified a GCC2-anaplastic lymphoma kinase (ALK) fusion protein in an NSCLC patient [27]. Overexpression of GCC2-ALK has been shown to activate downstream ALK signaling that can be inhibited by ALK inhibitors including crizotinib and ceritinib; therefore, the direct involvement of GCC2 makes it a promising target for NSCLC diagnosis and/or treatment [28].

In this study, we report on the use of GCC2 as a specific biomarker to distinguish between cancer-specific exosomes and normal cell-derived exosomes isolated from the body fluids of NSCLC patients, which can improve the diagnosis of cancer.

## 2. Materials and Methods

### 2.1. Patient Samples

Blood samples were collected from patients at Korea University Guro Hospital and Korea University Anam Hospital, Seoul, Korea. The subjects included 16 healthy controls (10 males and 6 females; mean age 26.3 ± 2.4 years) and 70 patients (35 males and 35 females; mean age 63.3 ± 11.0 years) who were scheduled to undergo lobectomies with a mediastinal lymph node dissection by video-assisted thoracoscopic surgery. The histological type and stage of lung cancer are listed in Table 1. Patients who received neoadjuvant chemotherapy before surgery were excluded.

Peripheral blood samples (3 mL) were drawn from healthy control subjects and patients with lung cancer. The samples were collected in EDTA-coated tubes and centrifuged at 3000 rpm for 15 min at 4 °C. The plasma layer was carefully removed without disturbing the buffy coat, transferred to a fresh tube, and then stored at −80 °C until use. A 0.5-mL aliquot of the blood plasma was centrifuged at 15,000× *g* for 10 min at 4 °C to remove residual cells, cell debris, apoptotic bodies, and nuclei. Plasmas were isolated from blood samples because of the variability of exosomal protein levels in serum caused by blood clot formation [29,30]. The clinical study protocol was approved by the Institutional Review Boards of the Korea University Guro Hospital (2020GR0340) and the Korea University Anam Hospital (2017AN0386). This research was performed in accordance with the principles of the Declaration of Helsinki. Informed consent was obtained from all subjects, who were over the age of 18 years.

### 2.2. Exosome Isolation from Plasma and Cell Culture Medium

A 0.5-mL aliquot of blood plasma was loaded into a disposable 10-mL column (Thermo Scientific Pierce, Waltham, MA, USA) made of porous Sepharose beads (Sephacryl S-200, Sepharose CL-6B, GE Healthcare Life Sciences, Chicago, IL, USA), in accordance with the manufacturer’s instructions. Exosomes were isolated from fractions, coinciding with a 30–100-nm size range, that were selected through nanoparticle tracking analysis (NTA) using a NanoSight NS300 device (Malvern Instruments, Malvern, UK). Each experiment was performed in triplicate (each with a 60-s capture time), and data were analyzed using the NanoSight NTA 2.3 Analytical Software (Malvern Instruments, Malvern, UK).

Human pulmonary alveolar epithelial cells (HPAEpiC) were purchased from ScienCell Research Laboratories and cultured in AEpiCM (ScienCell Research Laboratories, Carlsbad, CA, USA) with growth supplement (EpiCGS), 10% fetal bovine serum (FBS), and 1% penicillin/streptomycin (P/S) following the manufacturer’s instructions. NSCLC cell lines A549, PC9, H1299, H522, and H1650 were cultured in RPMI medium (HyClone, Logan, UT, USA) supplemented with 5% FBS (HyClone, Logan, UT, USA) and 1% P/S (Gibco, Waltham, MA, USA). The FBS used in this experiment was depleted of exosomes by ultracentrifugation at 120,000 × *g* for more than 12 h at 4 °C.

In vitro cell culture medium was collected from cells grown to 70% confluency for 48 h. After sequential centrifugation at 500× *g*, for 10 min and 5000× *g* for 30 min at 4 °C to remove cell debris and intact cells, the supernatant was collected and filtered through a 0.22-µm filter. Subsequently, it was concentrated using an Amicon^®^ Ultra-100K filter (Merck Millipore, Burlington, MA, USA) according to the manufacturer’s instructions. The concentrated supernatant was purified using a size exclusion chromatography (SEC) kit (EXoPERT Inc., Seoul, Korea); eluted fractions #6–8 (0.5 mL each) were collected. Aliquots containing the exosomes were assessed by NTA (Malvern Instruments, Malvern, UK) to determine the particle size.

### 2.3. Transmission Electron Microscopy (TEM)

All samples were mixed with an equal volume of 4% paraformaldehyde. Subsequently, 5 µL of the mixed solution was deposited onto Formvar carbon-coated grids and left for 15 min to allow for absorption into the membranes. The grids were then washed with phosphate-buffered saline (PBS) droplets and incubated along with drops of 2.5% glutaraldehyde for 1 min, followed by eight washes with distilled water drops. For contrast samples, phosphotungstic acid solution was dropped onto the grid at a pH of 7 for 5 min, after which the remaining solution was removed using a filter paper. After drying, the grids were observed under a transmission electron microscope at 200 kV. For immunogold labeling, TEM specimens were prepared by dropping 15 µL of the samples onto the TEM grid. After 10 min, the grid was washed three times with PBS droplets to remove residual substances. Then, the grid was immersed in 4 µg/mL of anti-CD63 antibody solution (Santa Cruz Biotechnology, Dallas, TX, USA) for 2 h and washed again with PBS droplets. The anti-mouse IgG–gold nanoparticle solution was diluted with PBS (1:20 *v*/*v*), in which the grid was immersed for 1 h, and then washed with PBS. Subsequently, the grid was fixed in 2.5% glutaraldehyde and then washed with distilled water drops. The residual solution was removed using a paper wipe and the grid was thoroughly dried.

### 2.4. Western Blotting

Proteins were extracted from cultured cells and exosomes using the radioimmunoprecipitation assay buffer (Thermo Scientific, Waltham, MA, USA) along with the Halt™ Protease Inhibitor Cocktail (Thermo Scientific, Waltham, MA, USA). Lysates were centrifuged at 15,000 rpm for 10 min at 4 °C, and the supernatants were collected in fresh tubes.

Protein concentrations were measured using the Bradford assay, wherein the appropriate amount of each sample was boiled with 5X SDS-PAGE sample buffer (Thermo Scientific, Waltham, MA, USA). Samples were separated on 6 and 12% SDS acrylamide gels and then transferred onto a PVDF membrane. The membrane was fixed with 5% skimmed milk in Tris-buffered saline containing 0.1% Tween20 for 1 h, followed by overnight incubation in diluted primary antibody solutions for CD63 (1:250; SC-15363, Santa Cruz Biotechnology, Dallas, TX, USA), CD9 (1:500; SC-13118, Santa Cruz Biotechnology, Dallas, TX, USA), CD81 (1:500; bs-6943R, Bioss, Woburn, MA, USA), CD31 (1:500; SC-376764, Santa Cruz Biotechnology, Dallas, TX, USA), and GCC2 (1:250; SC-242898, Santa Cruz Biotechnology, Dallas, TX, USA). Subsequently, the membrane was incubated for 1 h at room temperature with horseradish peroxidase-conjugated secondary antibodies at a dilution of 1:10,000. Detailed information about western blot can be found at Supplementary Figure S4.

### 2.5. Proteomics Analysis

The exosome lysates were fractionated on a 4–12% gel using SDS-PAGE. Subsequently, the gels were diced into pieces smaller than 1 mm^2^ and rinsed with 200 µL of water, and then rinsed twice with 200 µL of 25 mM ammonium bicarbonate in 50% acetonitrile followed by a final rinse in 100 µL of acetonitrile to dehydrate the gel plugs, which were then lyophilized. The dry gel plugs were rehydrated in 150 µL of 25 mM ammonium bicarbonate (pH 8, with 12.5 ng/µL trypsin). After rehydration, the gel plugs were incubated overnight at 37 °C in ammonium bicarbonate (100 µL, 25 mM). Digested samples were desalted using a C_18_ micro spin column (Harvard Apparatus, Cambridge, MA, USA). The pooled extracts were reduced to dryness and reconstituted in 80% acetonitrile/0.1% formic acid for subsequent trapped ion mobility spectrometry coupled with time-of-flight mass spectrometry (timsTOF MS). Samples were analyzed on a nanoElute (Bruker, Billerica, MA, USA) coupled with a timsTOF Pro (Bruker, Billerica, MA, USA) equipped with a CaptiveSpray source. Peptides were separated on a 25 cm × 75 µm analytical column (1.6-µm C_18_ beads packed into an emitter tip; IonOpticks, Victoria, Australia) with a linear gradient of 2–95% of solvent B (100% acetonitrile/0.1% formic acid) over a 120-min gradient at a constant flow (0.4 µL/min). The column was maintained at 50 °C. The timsTOF Pro was operated in PASEF mode using Compass HyStar 5.0.37.1. Six samples were analyzed each day using the following settings: mass range 100–1700 *m/z*, 1/K_0_ 0.6–1.3 V·s/cm^2^, ramp time 100 ms, lock duty cycle 100%, capillary voltage 1600 V, dry gas 3.0 l/min, dry temperature 180 °C; PASEF settings: 10 MS/MS scans (total cycle time 1.16 s), charge range 0–5, an active exclusion for 0.4 min, scheduling target intensity 20,000, intensity threshold 2500, CID collision energy 10 eV.

Data files were uploaded to PEAKS X (Bioinformatics Solutions, Waterloo, ON, Canada) for *de novo* sequencing and database search. The sequences were searched against the UniProt database (downloaded January 2019; 34,064 entries); the mass error tolerances for parent and fragment ions were set to 20 ppm and 0.05 Da, respectively. Trypsin enzyme specificity and acetylation (protein N-terminal), methionine oxidation, and phosphorylation (STY) were selected as variable modifications. False discovery rate (FDR) estimation was enabled, and the peptides were filtered at a 1% FDR at the peptide-spectrum match level. Protein filtering was disabled by setting protein −log_10_ [P] scores for two unique peptides as a requirement for significant peptides.

### 2.6. Quantitative Real-Time PCR (qRT-PCR)

Total RNA was extracted using TRI reagent (Invitrogen, Waltham, MA, USA) according to the manufacturer’s instructions. cDNA was synthesized using reverse transcriptase (Roche, Basel, Switzerland) with 5 µg of total RNA and oligo dT primers. qRT-PCR was performed in triplicate using the KAPA SYBR FAST ABI Prism qPCR kit reagents (KAPA Biosystems, Burlington, MA, USA). Primers for the genes of interest were synthesized by Cosmogenetech (Seoul, Republic of Korea). GCC2 and GAPDH were amplified the following specific primers: GCC2-F, 5′-AAA CCT CTG CGG AAC AGC ACC A-3′; GCC2-R, 5′-GAA CTC GGA CTT TGT AGC TCT CG-3′; GAPDH-F, 5′-GCT CAG ACA CCA TGG GGA AGG T-3′; GAPDH-R, 5′-GTG GTG CAG GAG GCA TTG CTG A-3′.

### 2.7. Enzyme-Linked Immunosorbent Assay (ELISA) for Exosomal GCC2 Detection

A GCC2 ELISA assay was performed using the GCC2 ELISA kit (MyBioSource, San Diego, CA, USA) according to the manufacturer’s instructions.

### 2.8. Statistical Analysis

Differences in exosomal GCC2 concentration between the groups were assessed using a one-way ANOVA, followed by the Scheffe or *t*-test. Kruskal–Wallis and Jonckheere–Terpstra tests were used to evaluate the trend for exosomal GCC2 concentrations at different pathological stages. Statistical analysis was performed using IBS SPSS Statistics 22.0 (IBM Corp., Armonk, NY, USA) and MedCalc 19.0.3 (MedCalc Software, Mariakerke, Ostend, Belgium) in consultation with the Medical Statistical Consulting Center of Korea University Guro Hospital. Bar graphs were generated using GraphPad Prism 7 (GraphPad Software, San Diego, CA, USA). Data are expressed as mean ± SD.

## 3. Results

### 3.1. Isolation of Exosomes by SEC

High-quality exosome purification is crucial for basic research and various diagnostic applications. Exosomes were isolated from pre-cleared cell culture media and plasma samples by centrifugation and SEC, as reported in our previous studies [31,32,33]. NTA was performed to determine the sizes and concentrations of exosomes in the eluted fractions. Fractions containing abundant concentrations of vesicles with similar sizes as exosomes (30–150 nm) were pooled and used as an exosome solution.

**Figure 1:**
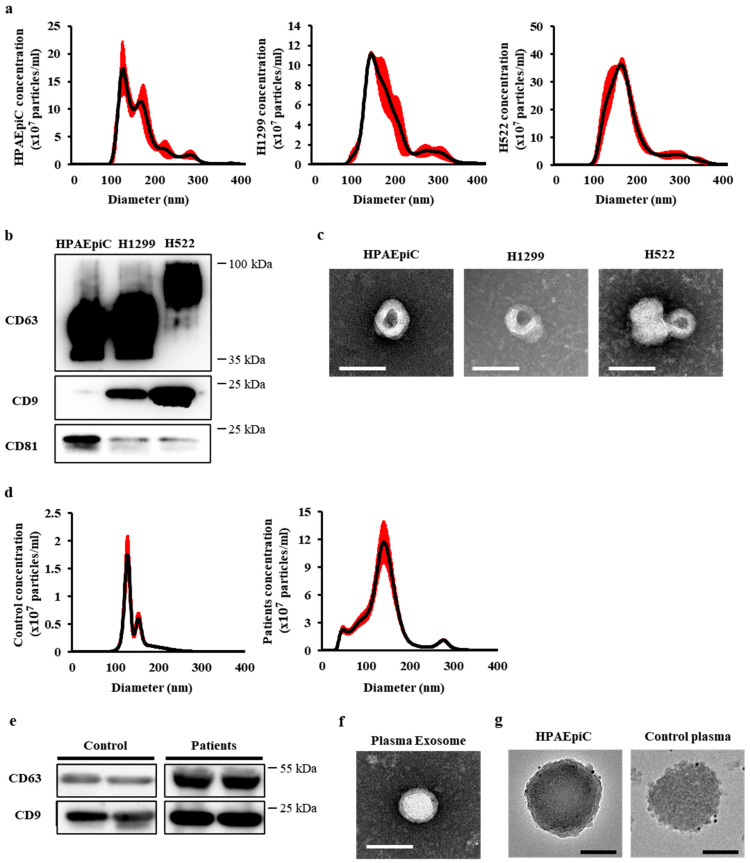
Characterization of exosomes derived from normal and lung cancer cell lines. (**a**) Average size of SEC fractions 6–8 determined by NTA. Average sizes of SEC fractions 6, 7, and 8 were 134.131 ± 1.99, 138.73 ± 6.98, and 131 ± 3.2 nm (mean ± SD), respectively. (**b**) Western blot analysis of the CD63, CD9, and CD81 exosomal proteins in the exosome lysates (6 µg of total protein/well of acrylamide gel). (**c**) TEM analysis shows the typical cup shape of the exosomes. Scale bar = 100 nm. (**d**) Average size of SEC fractions 6–8 from healthy and patient plasmas determined by NTA were 135.36 ± 5.31 and 138.71 ± 12.40 (mean ± SD), respectively. (**e**) Western blot analysis of the CD63 and CD9 exosomal proteins in the exosomal lysates. (**f**) TEM analysis shows the typical cup shape of the exosomes derived from plasma. Scale bar = 100 nm. (**g**) TEM analysis shows immunogold labeling using an anti-CD63 antibody for the exosomes isolated from HPAEpiC and human plasmas. Scale bar = 100 nm.

NTA revealed the concentration and size distribution profiles of the pooled exosome-rich fractions, which were mostly within the 30–150 nm size range (Figure 1a,d). The exosome concentrations in healthy control and patient groups were 9.9 × 10^8^ ± 8.4 × 10^8^ particles/mL and 5.3 × 10^9^ ± 9.4 × 10^9^ particles/mL, respectively, which is an approximate 5.8-fold increase in exosome concentration in patients compared to that in healthy controls (*p* < 0.0001). Western blotting demonstrated that the exosomes were immunoreactive to exosome-specific markers CD63, CD9, and CD81 (Figure 1b,e). TEM examinations revealed a typical exosome-like cup-shaped morphology with average diameter of approximately 100 nm (Figure 1c,f). The exosomes derived from HPAEpiC and healthy control plasmas were positive against the anti-CD63 antibody with gold nanoparticle tagged secondary antibody, as revealed by TEM analysis (Figure 1g).

Exosomal particles (10 µg) isolated by SEC were lysed and loaded onto a gel for SDS-PAGE, as shown in our previous studies [31,34]. The detection of CD63 and CD9 in exosomal proteins obtained from NSCLC cell lines was more distinct than that from HPAEpiC; however, overall, CD9 was more expressed than CD81 in the lung cell lines (Figure 1b). Here, we established a high-quality purification method for exosomes, ranging from 30 to 150 nm in size.

### 3.2. Identification of GCC2 as a Potential Diagnostic Marker in Various NSCLC Cell Lines

**Figure 2:**
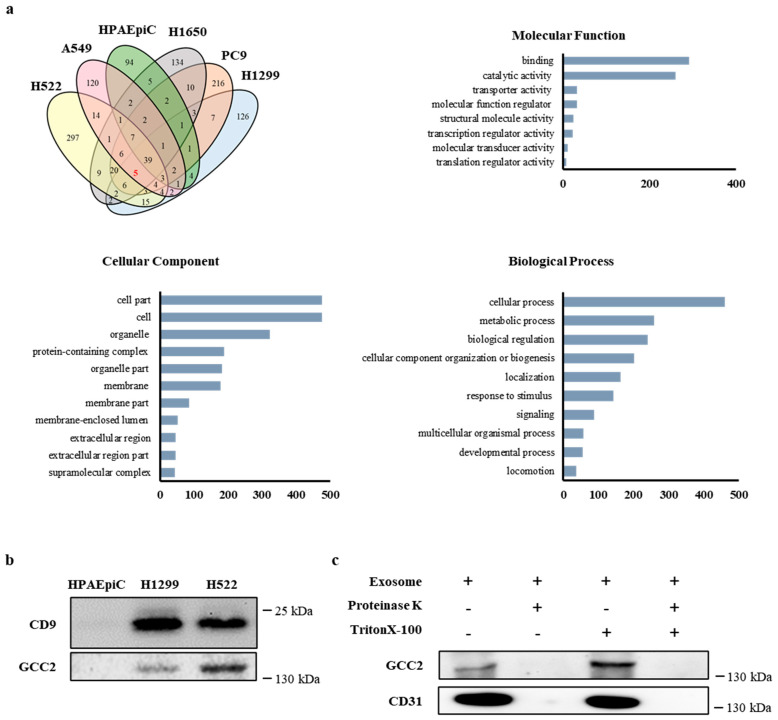
Identification of a potential factor for NSCLC diagnosis and treatment by proteomic analysis. (**a**) A Venn diagram for the proteomic analysis and Gene Ontology classification of the exosomes derived from six cell lines. (**b**) GCC2 expression in the exosomal lysates determined by western blotting. (**c**) GCC2 protein present on both the surface and inside the exosomes. The GCC2 protein disappeared following the exposure of the exosomal surface proteins to proteinase K.

We analyzed the exosomes derived from the following cell lines: HPAEpiC, H1299, H522, A549, PC9, and H1650. The NSCLC exosomal markers were identified by selecting cancer cell lines that contained mutations in *KRAS*, *EGFR*, and *tumor protein p53* (*TP53*). H1299 has a neuroblastoma *RAS (NRAS)* mutation; H522 has a *TP53* mutation; A549 has a *KRAS* mutation; and PC9 and H1650 have *EGFR* mutations. Normal human pulmonary alveolar epithelial cells (HPAEpiCs) were used as the control cells. Protein compositions of exosomes isolated from the cell lines (HPAEpiC, H1299, H522, A549, PC9, H1650) were determined by proteomics analyses. The number of abundant exosomal proteins totaled 165 in HPAEpiC, 231 in H1299, 436 in H522, 338 in PC9, 214 in A549, and 258 in H1650 (Supplementary Table S1). In order to identify the exosomal proteins obtained from the lung cancer cell lines, the exosomal proteins from each cell line were grouped and compared with those from normal HPAEpiCs (Figure 2a). TUBA1C, GAPDH, KRT25, GCC2, and POTEKP were the five proteins identified as potential lung cancer-specific exosome biomarkers. These proteins were annotated with gene ontology (GO) terms using PANTHER (http://www.pantherdb.org, accessed 1 March 2016). Most of these proteins are involved in cellular processes and cellular component organization or biogenesis.

We selected GCC2, a trans-golgi network (TGN) membrane protein that tethers vesicles containing mannose 6-phosphate receptors, which are inbound from late endosomes to the TGN [35]. According to the Human Protein Atlas, GCC2 can be used as a prognostic marker in liver cancer, albeit with low confidence (http://www.proteinatlas.org, accessed 1 March 2016). In order to examine whether GCC2 could be used as a potential biomarker for lung cancer, we tested the RNA and protein expression levels in the different cell lines. The qRT-PCR analysis revealed that expression levels of the *GCC2* gene in H1299 and H522 were fourfold higher than in HPAEpiC and the other cancer cell lines. This suggests that *GCC2* expression is likely to be upregulated in specific lung cancer cell types (Supplementary Figure S1a).

Next, to examine whether the GCC2 protein is expressed in the cancer cell line-derived exosomes, we analyzed GCC2 protein levels in the exosomal lysates of H1299, H522, and HPAEpiC cells. The GCC2 protein was expressed in the exosomal lysates of H1299 and H522; however, it was barely expressed in the HPAEpiCs (Figure 2b). Interestingly, the exosome concentrations in lung cancer patient-derived plasmas increased with the progression of the pathological stage (Supplementary Figure S1c). Western blotting was performed to ascertain the presence of the GCC2 protein on the surface of the exosomes. Digestion of patient-derived exosomal lysates with proteinase K resulted in the complete degradation of the GCC2 protein; this was compared with no proteinase K treatment, as shown in lanes 1 and 2 of Figure 2c. The membrane protein CD31 was used as a control. Furthermore, when the exosomal lysates were treated with the Triton X-100 detergent (lanes 3 and 4), the GCC2 protein levels increased notably compared to those in the untreated sample (lane 1). Collectively, these results indicated that the presence of GCC2 on the surface of the exosomes could be a predictive marker for lung cancer diagnosis, suggesting that GCC2 could be a potential biomarker for NSCLC.

### 3.3. Verification of GCC2 as a Specific Biomarker for Early Lung Cancer Diagnosis in Patients with NSCLC

**Figure 3:**
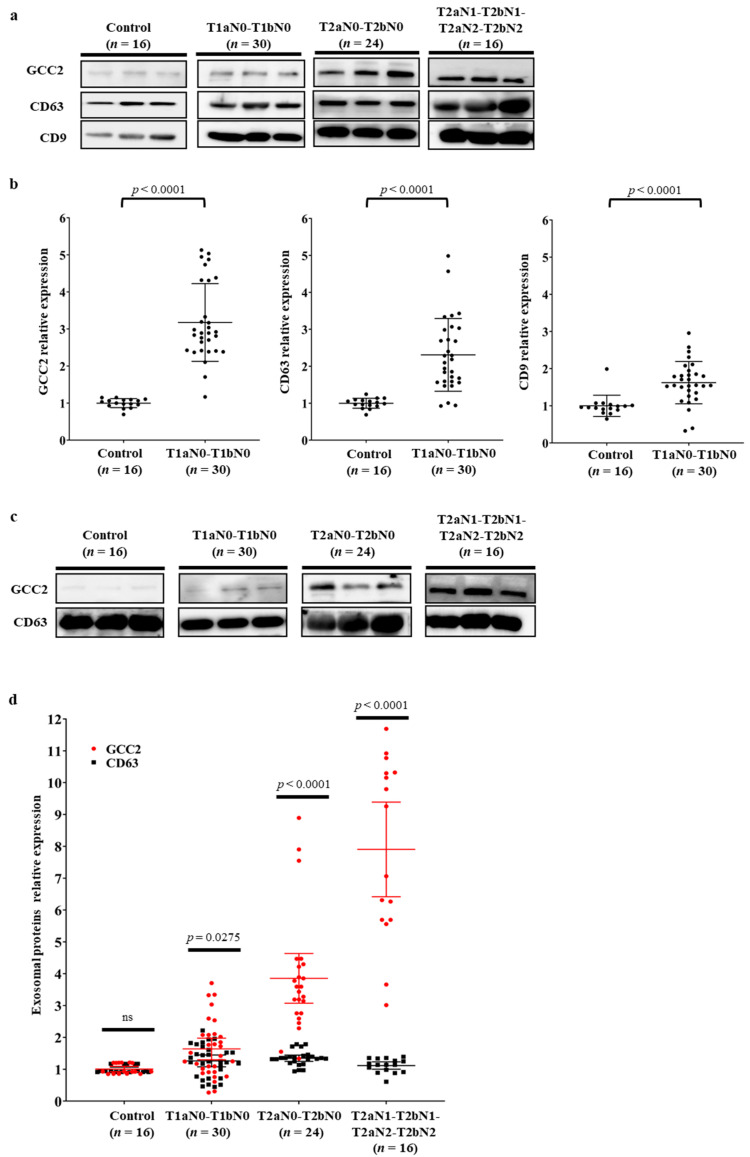
GCC2 levels in NSCLC patient plasma-derived exosomes. (**a**) Comparison of GCC2, CD63, and CD9 protein levels in the plasma-derived exosomes isolated from the healthy control group (*n* = 16) and patients suffering from different pathological stages of NSCLC (*n* = 70) by western blot analysis. Each of the three lanes represents 3 healthy controls and 3 patients at different pathological stages of NSCLC. (**b**) Comparison of the relative intensities of GCC2, CD63, and CD9 protein levels between the control and T1aN0-T1bN0 patient groups (*n* = 30). Protein intensities were measured using ImageJ. An independent Student’s *t*-test and the Jonckheere–Terpstra test were used for statistical validation. (**c**) Comparison of GCC2 and CD63 protein levels in the plasma-derived exosomes isolated from the control group and patients suffering from different pathological stages of NSCLC by western blotting. The same exosome number determined by NTA was used for each sample during western blotting. Each of three lanes represents 3 healthy controls and 3 patients at different pathological stages of NSCLC. (**d**) Comparison of the relative intensities of GCC2 and CD63 protein levels. The GCC2 levels, but not CD63 levels, gradually increased as the pathological stage progressed.

We performed western blot analysis on the exosomes isolated from lung cancer patients (*n* = 70) diagnosed with NSCLC adenocarcinoma stage T1aN0-T1bN0 (*n* = 30, 42.9%), T2aN0-T2bN0 (*n* = 24, 34.3%), and T2aN1-T2bN1-T2aN2-T2bN2 (*n* = 16, 22.9%), and normal controls (*n* = 16) without a cancer diagnosis (Table 1). GCC2 expression was very weak in the normal group; however, it gradually increased as the pathological stage of lung cancer progressed (Figure 3a). The exosomal markers CD63 and CD9 were used to standardize the GCC2 expression levels at different pathological stages. Surprisingly, the GCC2 protein levels in patients with early-stage lung cancer (T1aN0-T1bN0) increased by more than 3.2-fold (*p* < 0.0001) compared with those of the normal group. However, the CD63 and CD9 proteins levels were not significantly higher in the early stage group than those in the normal group (Figure 3b).

Supplementary Figure S2 displays the GCC2 levels for the normal group and the patient group at different pathological stages. The relative intensity values were 5.4- (*p* < 0.0001) and 6.7-fold (*p* < 0.0001) for T2aN0-T2bN0 and T2aN1-T2bN1-T2aN2-T2bN2, respectively. However, the relative intensities of CD63 and CD9 were increased by 2.8- (*p* < 0.0001) and 2.0-fold (*p* < 0.0001) for T2aN0-T2bN0, and 4.9- (*p* < 0.0001) and 3.3-fold (*p* < 0.0001) for T2aN1-T2bN1-T2aN2-T2bN2, respectively. Based on the pathological stage progression, the most significant *p*-value was obtained for CD63 (trend test *p*-value < 0.0001 vs. CD9 trend test *p*-value < 0.001).

Therefore, in order to precisely examine GCC2 protein levels, we used NTA to ensure the same number of exosome particles (1.0 × 10^9^ particles/mL) were present in samples obtained at all pathological stages, followed by western blotting. As shown in Figure 3c,d, unlike CD63 levels, the GCC2 levels increased gradually as the pathological stages progressed.

**Figure 4:**
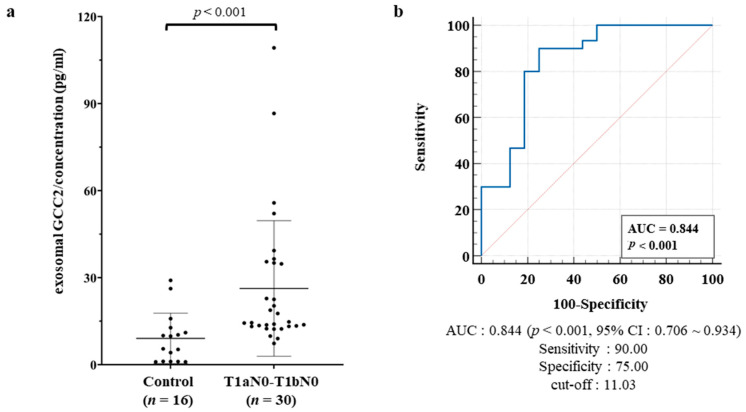
Evaluation of the exosomal GCC2 protein as a diagnostic biomarker for patients with early-stage lung cancer. (**a**) Exosomal GCC2 protein concentrations in the healthy control group (*n* = 16) and patients with T1aN0-T1bN0 lung cancer (*n* = 30) determined by ELISA. (**b**) ROC curve based on the exosomal GCC2 protein levels to distinguish patients with early NSCLC from normal healthy controls. An independent Student’s *t*-test and the Jonckheere–Terpstra test were used for statistical validation.

Next, we quantified the exosomal GCC2 protein levels in early-stage lung cancer patients’ plasmas using ELISA. Results (Figure 4a) revealed that the GCC2 protein concentrations were significantly higher (normal 9.07 pg/mL vs. patients’ 26.28 pg/mL, *p* < 0.001) in early-stage lung cancer patients (T1aN0-T1bN0; *n* = 30) than in the normal controls (*n* = 16). In comparison to that in normal controls, the GCC2 protein concentration increased by 3.97-fold (normal 9.07 pg/mL vs. patients 35.99 pg/mL, *p* < 0.001) in the middle stage (T2aN0-T2bN0) and 7.10-fold (normal 9.07 pg/mL vs. patients 64.40 pg/mL, *p* = 0.0136) in the late stage (T2aN1-T2bN1-T2aN2-T2bN2) (Supplementary Figure S3a,c).

Subsequently, a receiver operating characteristic (ROC) curve was constructed to assess the diagnostic value of the exosomal GCC2 protein for patients with early-stage lung cancer. The sensitivity and specificity values for exosomal GCC2 were 90.00% and 75.00%, respectively, with an area under the curve (AUC) of 0.844 (95% confidence interval [CI]: 0.706–0.934, *p* < 0.001), and a cut-off of 11.03 pg/mL (Figure 4b). The sensitivity and specificity values of exosomal GCC2 were 75.00 and 87.50%, respectively, with an AUC of 0.849 (95 % CI: 0.700–0.942, *p* < 0.001) for the middle stage (T2aN0-T2bN0), and 81.25 and 87.50%, respectively, with an AUC of 0.879 (95% CI: 0.72–0.96, *p* < 0.001) for the late stage (T2aN1-T2bN1-T2aN2-T2bN2) (Supplementary Figure S3b,d).

These results revealed that GCC2 protein levels in the lung cancer patient groups were significantly higher than those in the normal group. Notably, the GCC2 levels were significantly high in patients with early-stage (T1aN0-T1bN0) lung cancer, which suggests that plasma-derived exosomal GCC2 protein level could be a feasible, valuable, and non-invasive biomarker for the early diagnosis of lung cancer.

## 4. Discussion

The detection of early-stage NSCLC can reduce relapse and mortality rates [1,36]; therefore, a new early diagnostic biomarker could become a prerequisite for NSCLC treatment. Currently, surgical resection of tumors is possible in only 20% of patients with NSCLC because of late diagnosis [37]. Recent studies have suggested the diagnostic potential of exosomal proteins for NSCLC [38,39]. Several advantages of exosomes, including easy acquisition, isolation, and storage, make them ideal biomarkers. Although exosome isolation may be easy, it is time-consuming because it requires significant quality control, even with the advantages of liquid biopsies [40,41].

Here, we isolated and characterized exosomes derived from normal and cancer cell lines, as well as from the plasma of healthy individuals and patients suffering from different pathological stages of NSCLC. The number and size distributions of exosomes were quantified by western blotting and TEM analysis, respectively (Figure 1). Interestingly, the number (Supplementary Figure S1c), not the average size of the secreted exosomes (Supplementary Table S2), was significantly different between normal individuals and early-stage lung cancer patients. These results suggest that an increase in the number of exosomes in patient plasma could be a potential marker for cancer diagnosis.

We identified an exosomal protein, GCC2, through proteomic analysis of exosomal proteins extracted from five cancer cell lines (Figure 2). GCC2 is a peripheral membrane protein localized to the TGN that interacts with many other proteins and has diverse functions [26,35,42]. Although GCC2 is expressed in most cancer cell types, it exhibits relatively moderate cytoplasmic and membranous immunoreactivity in most cancers, including lung cancer [43].

Interestingly, the GCC2 protein levels in exosomes isolated from cancer cell lines and patient plasma at different NSCLC pathological stages were dramatically higher than those of the respective controls (Figure 2b and Figure 3). Compared to that in the normal group, the GCC2 protein level increased progressively in the patients as the pathological stages of lung cancer progressed (Supplementary Figure S2), indicating that GCC2+ exosomes could be a reliable biomarker to detect NSCLC. These results suggest that NSCLC-derived exosomes are enriched with the GCC2 protein. The GCC2+ exosomes can serve as a safe prognostic marker compared to computed tomography, which is risky, requires several exposures, and is expensive for patients. Although the mechanism by which the amount of GCC2 protein increases in patient exosomes has not yet been elucidated, as the severity of the disease increases, the increase in the amount of GCC2 protein observed in a single exosome could be a result of other unknown causes. It is likely that GCC2 is secreted along with the exosomes during the process of exosome secretion by increasing post-translational modifications, after transcription and translation of the GCC2 gene.

Surprisingly, the GCC2 protein levels in patient plasma were significantly high for early-stage (T1aN0-T1bN0) NSCLC (Figure 3a,b). We also evaluated the diagnostic capacity of the exosomal GCC2 protein in patients with early-stage lung cancer. Exosomal GCC2 displayed a sensitivity of 90.00% and a specificity of 75.00%, even though the accuracy was relatively higher in patients at more advanced pathological stages (Supplementary Figure S3d). Our ROC curve analysis showed that GCC2 has excellent diagnostic potential with an AUC of 0.844 (95% CI: 0.706–0.934, *p* < 0.001) when comparing healthy individuals and early-stage NSCLC patients. These results suggest that using GCC2 as a biomarker could provide accurate information to detect early-stage NSCLC. Recently, we reported that early-stage lung cancer could be diagnosed through deep-learning-based surfaced-enhanced Raman spectroscopy analysis of the circulating exosomes [33]. GCC2 is located on the exosome membrane; therefore, detecting GCC2+ exosomes using an antibody is easy, making it possible to diagnose lung cancer by analyzing the GCC2 exosome biomarker in the peripheral blood of the patients in the future. In contrast, methods to detect dsDNA in exosomes and their existence remain unclear [40]. Moreover, the pre-treatment process to detect lncRNA and miRNA in exosomes is complicated, and it is likely for the purity of a very small amount of RNA in exosomes to be low [44].

In conclusion, our results demonstrate GCC2 as an exosomal biomarker for the early-stage diagnosis of NSCLC. The ROC curve analysis revealed a significantly high sensitivity and specificity of exosomal GCC2 with a high AUC value (0.844) to discriminate between patients with early NSCLC from healthy controls. These results suggest that GCC2 can be an effective biomarker for early diagnosis of NSCLC. This pilot study has evaluated the diagnostic potential of exosomal GCC2 in a small number of patients, which is the main limitation of this study.

## 5. Conclusions

Our study suggested a new exosomal biomarker, GCC2, with high specificity and sensitivity in detecting early-stage NSCLC. We identified GCC2 through proteomic analysis of exosomes secreted from cell lines and obtained from patients’ plasma. Western blotting and ELISA revealed significant change in exosomal GCC2 depending on the pathological stage. GCC2 in peripheral blood exosomes for diagnosis is expected to greatly contribute to the detection of asymptomatic early-stage lung cancer patients during routine screening.

## Figures and Tables

**Table 1:** Summary of clinical characteristics of patients with non-small cell lung cancer.

		No. of Patients	Percentage (%)
Sex	Male/female	35/35	50.0/50.0
Age	Mean, range	63.3, 40~86	-
Histology	Adenocarcinoma	70	100
Site of primary tumor	Right upper lobe	25	35.7
	Right middle lobe	7	10.0
	Right lower lobe	17	24.3
	Left upper lobe	10	14.3
	Left lower lobe	10	14.3
	Right upper lobe with right lower lobe	1	1.4
Mode of operation	Lobectomy	58	82.9
	Segmentectomy	9	12.9
	Wedge resection	3	4.3
Invasion	Lymphatic	4	5.7
	Venous	3	4.3
	Lymphatic with venous	2	2.9
	Lymphatic, venous, and perineural	1	1.4
	None	60	85.7
p-Stage	T1aN0–T1bN0	30	42.9
	T2aN0–T2bN0	24	34.3
	T2aN1, T2bN1, T2aN2, T2bN2	16	22.9
ALK	Negative	69	98.6
	Positive	1	1.4
EGFR	Wild type	53	75.7
	E19 Deletion	6	8.6
	E20 Insertion	1	1.4
	L858R mutation	10	14.3
Tumor size (mm)	Mean ± SD	22.1 ± 12.2	-

## Data Availability

The data that support the findings of this study are available from the corresponding author, S.H., upon reasonable request.

## Supplementary Materials

The following are available online at https://www.mdpi.com/article/10.3390/cancers13215482/s1, Figure S1: GCC2 expression and exosome secretion in lung cancer cell lines and patients (a) Expression levels of cellular GCC2 mRNA. * *p* < 0.05, ** *p* < 0.01, *** *p* < 0.001 (*n* = 3, technical replicates). (b) Exosome numbers derived from HPAEpiC and cancer cell lines. Cells (1.0 × 10^5^, 35-mm dish) were seeded in a cell culture dish. After 2 days, exosomes were isolated and assessed by NTA. (c) Concentration of the exosomes in healthy controls and patients suffering from different pathological stages of lung cancer using NTA. An independent student’s *t*-test and the Jonckheere-Terpstra test were used for statistical validation. Figure S2: Relative expression levels of the exosomal GCC2, CD63, and CD9 proteins in healthy controls and patients suffering from different pathological stages of lung cancer by western blot analysis. An independent student’s *t*-test and the Jonckheere-Terpstra test were used for statistical validation. Figure S3: Exosomal GCC2 protein concentrations and ROC curve analysis of ELISA data for healthy controls and patients suffering from different pathological stages of lung cancer (a) Exosomal GCC2 protein concentrations in healthy controls (*n* = 16) and patients with middle-stage (T2aN0-T2bN0) lung cancer (*n* = 24). (b) ROC curve analysis of the exosomal GCC2 protein concentrations in patients with middle-stage (T2aN0-T2bN0) lung cancer. (c) Exosomal GCC2 protein concentrations in healthy controls (*n* = 16) and patients with late-stage (T2aN1-T2bN1-T2aN2-T2bN2) lung cancer (*n* = 16). (d) ROC curve analysis of the exosomal GCC2 protein concentrations in patients with late-stage (T2aN1-T2bN1-T2aN2-T2bN2) lung cancer. Figure S4: Original images of western blot. Table S1: List of the total proteins identified by proteomic analysis. Table S2: Size of exosomes derived from the lung cancer cell lines and patient plasmas.
