# Supplementary material for: GCC2 as a New Early Diagnostic Biomarker for Non-Small Cell Lung Cancer
Source: Cancers (Basel). 2021 Oct 31;13(21):5482. doi: 10.3390/cancers13215482 (PMC8582534; doi:10.3390/cancers13215482)
Supplement: Supplementary file 1 [file cancers-13-05482-s001.zip › cancers-1421116-Figure S4.pdf]

CD63

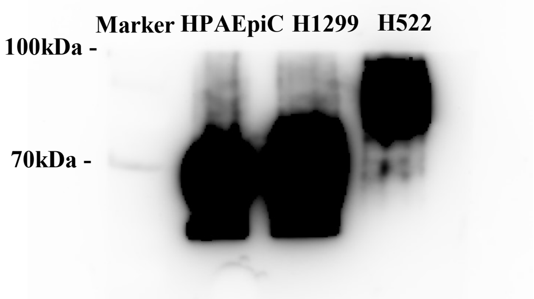

(Correspondence to Figure 1b in the manuscript)

CD9

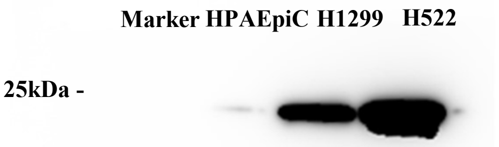

(Correspondence to Figure 1b in the manuscript)

CD81

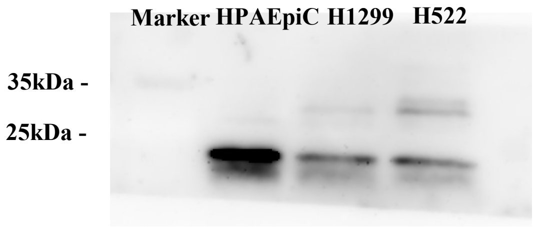

(Correspondence to Figure 1b in the manuscript)

CD9

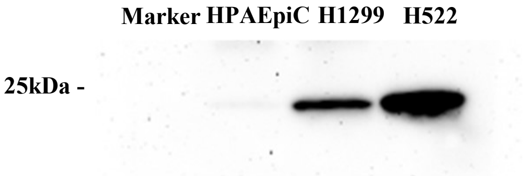

(Correspondence to Figure 2b in the manuscript)

GCC2

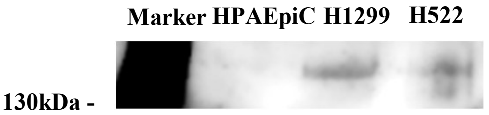

(Correspondence to Figure 2b in the manuscript)

GCC2

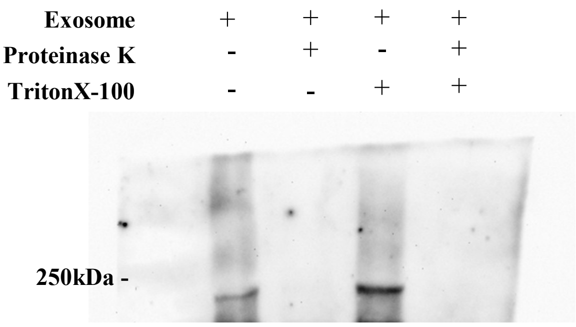

(Correspondence to Figure 2c in the manuscript)

CD31

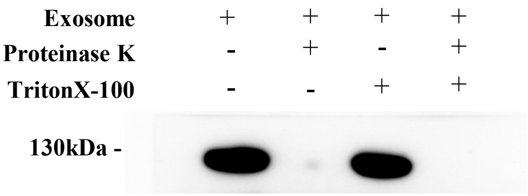

(Correspondence to Figure 2c in the manuscript)

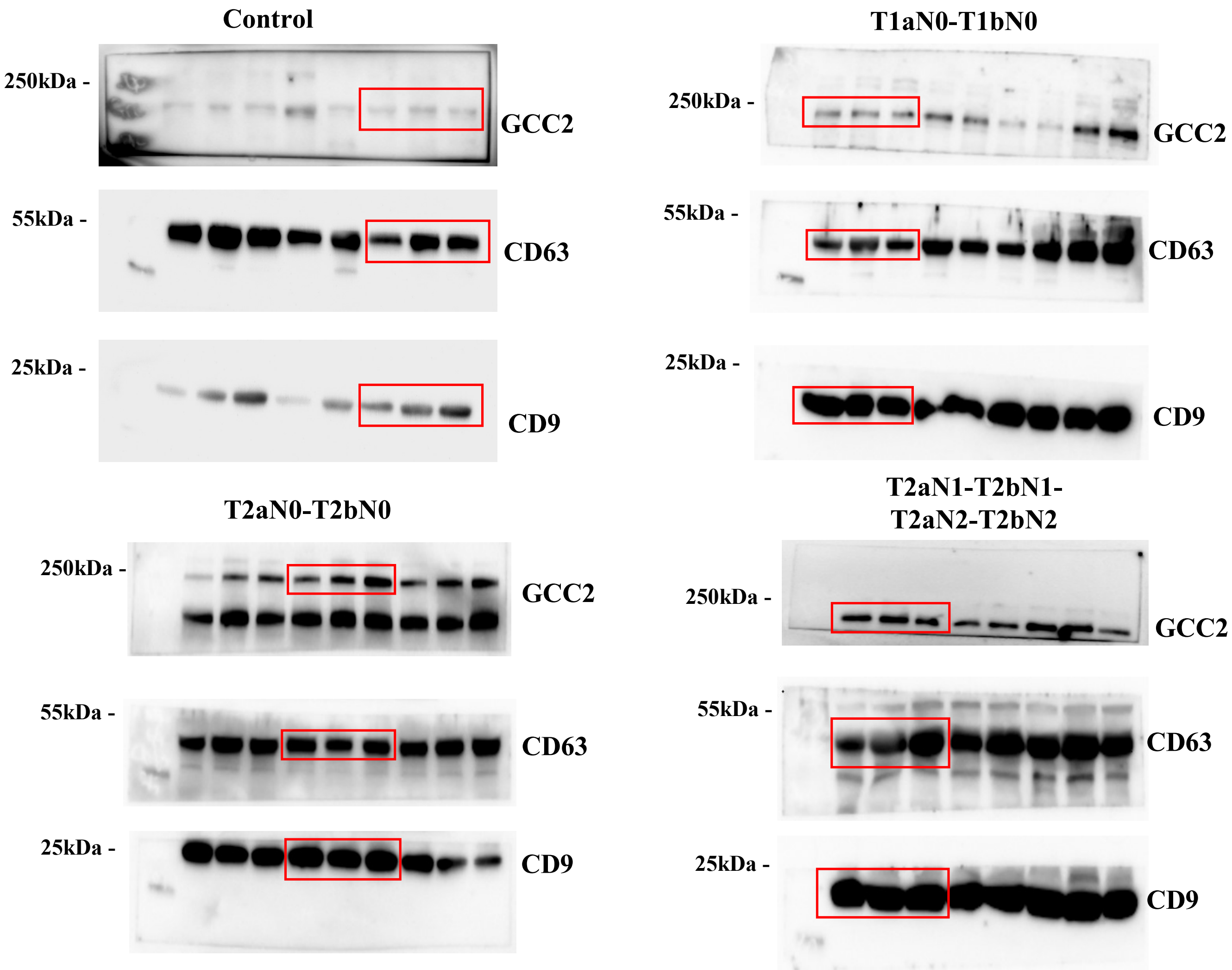

(Correspondence to Figure 3a in the manuscript)

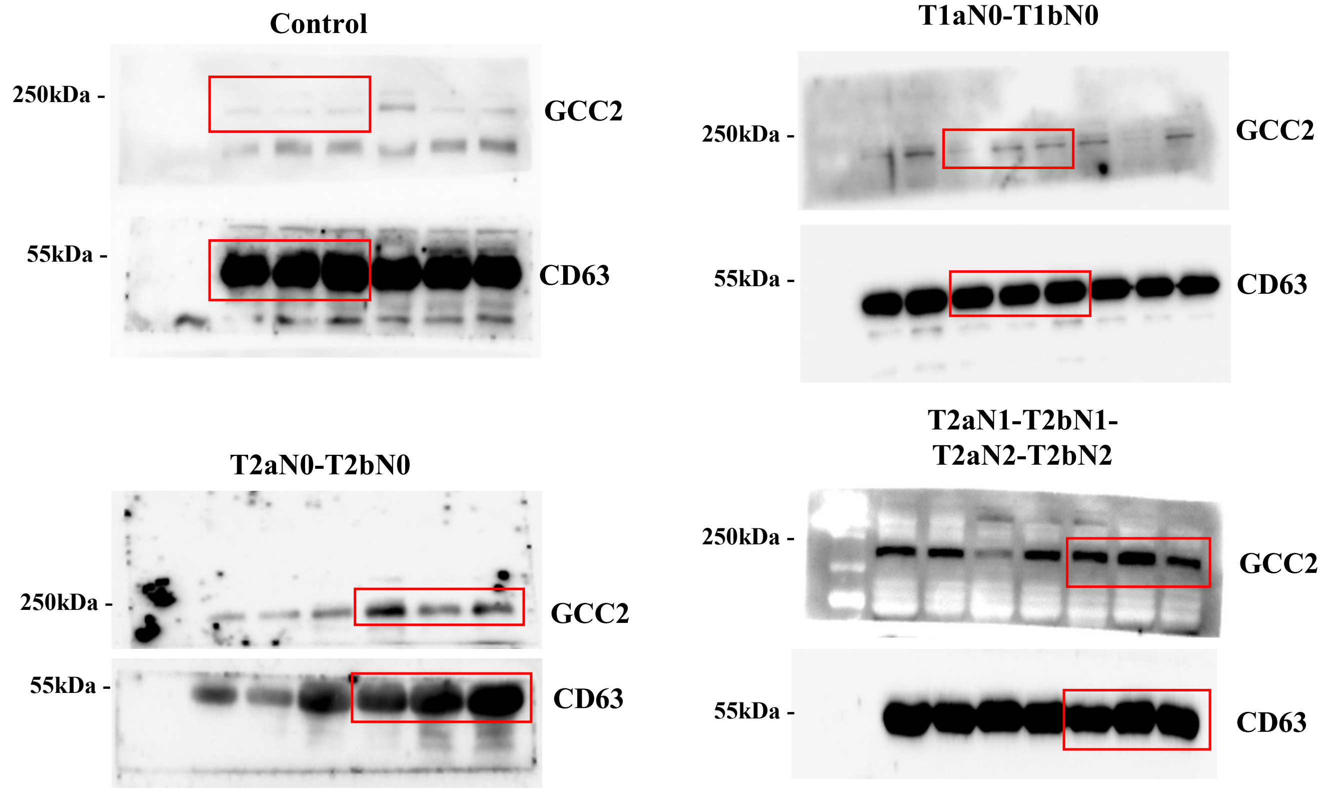

(Correspondence to Figure 3c in the manuscript)

Supplementary Figure S4. Original images of western blot.
