# Supplementary material for: GCC2 as a New Early Diagnostic Biomarker for Non-Small Cell Lung Cancer
Source: Cancers (Basel). 2021 Oct 31;13(21):5482. doi: 10.3390/cancers13215482 (PMC8582534; doi:10.3390/cancers13215482)
Supplement: Supplementary file 1 [file cancers-13-05482-s001.zip › cancers-1421116-supplementary.pdf]

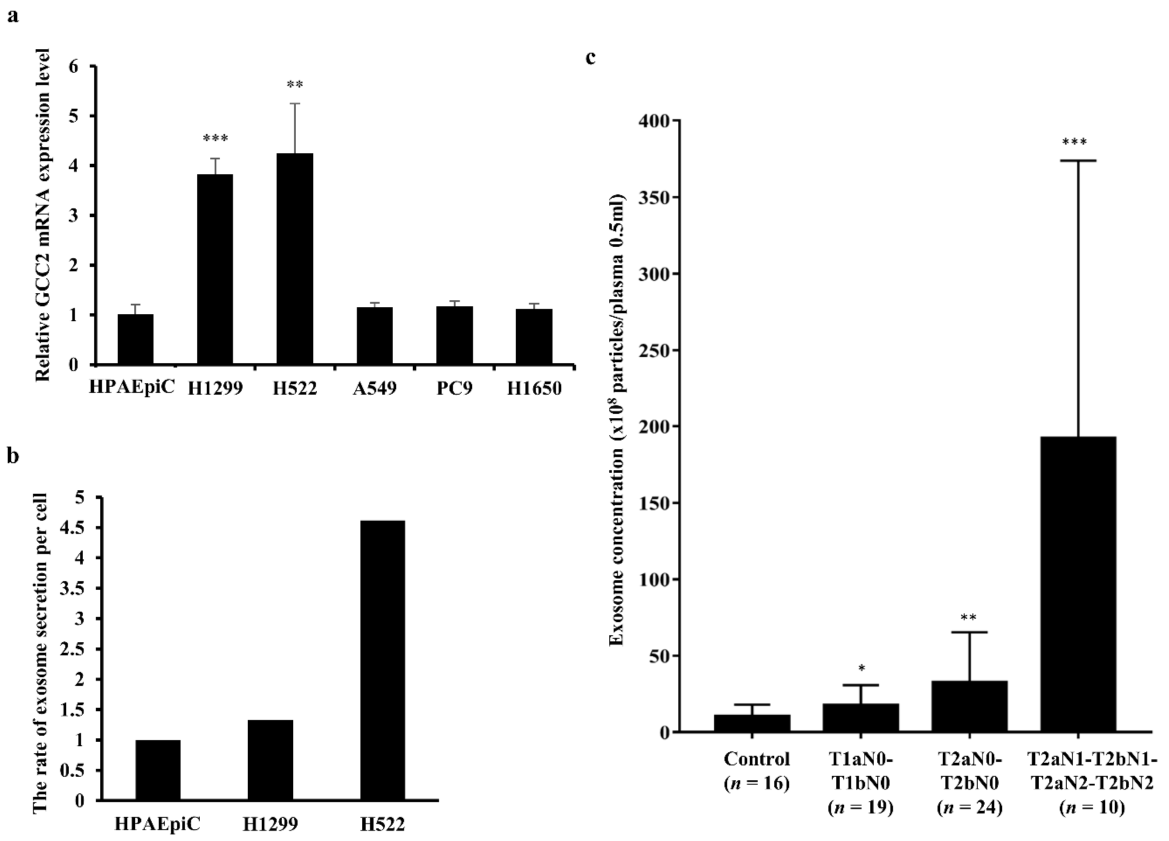

Figure S1. GCC2 expression and exosome secretion in lung cancer cell lines and patients (a) Expression levels of cellular GCC2 mRNA. * $p < 0.05$, ** $p < 0.01$, *** $p < 0.001$ ($n = 3$, technical replicates). (b) Exosome numbers derived from HPAEpiC and cancer cell lines. Cells (1.0×10^5, 35-mm dish) were seeded in a cell culture dish. After 2 days, exosomes were isolated and assessed by NTA. (c) Concentration of the exosomes in healthy controls and patients suffering from different pathological stages of lung cancer using NTA. An independent student's *t*-test and the Jonckheere-Terpstra test were used for statistical validation.

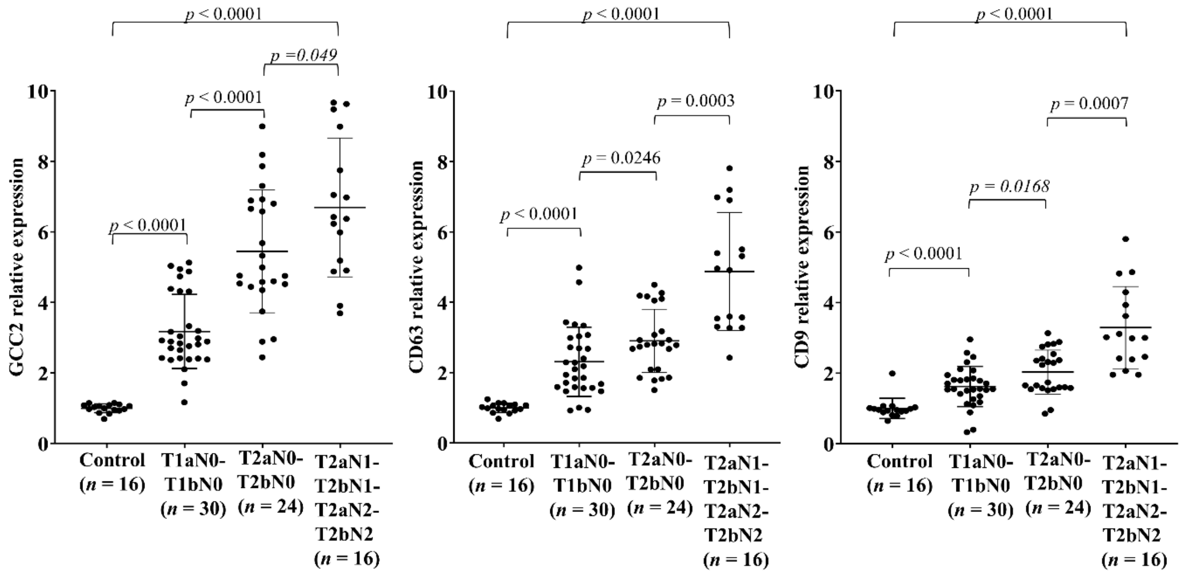

Figure S2. Relative expression levels of the exosomal GCC2, CD63, and CD9 proteins in healthy controls and patients suffering from different pathological stages of lung cancer by western blot analysis. An independent student's *t*-test and the Jonckheere-Terpstra test were used for statistical validation.

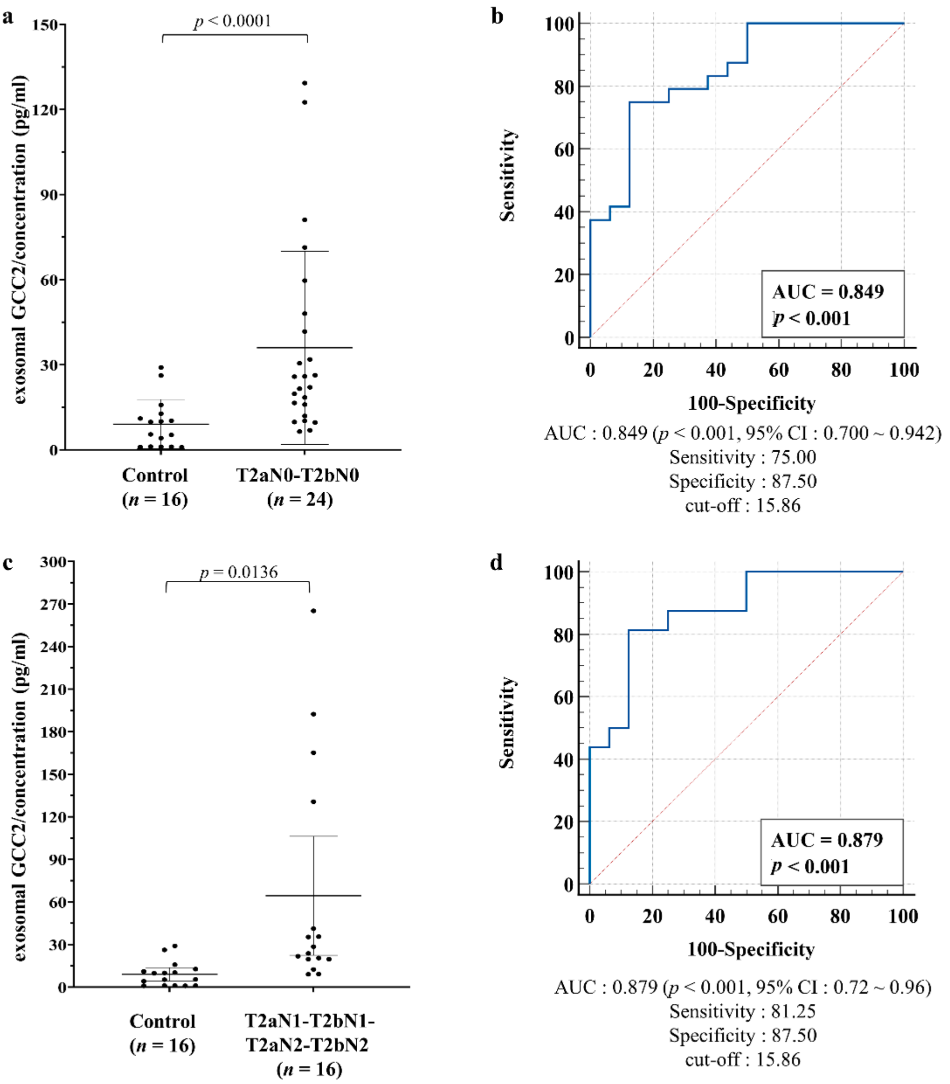

Figure S3. Exosomal GCC2 protein concentrations and ROC curve analysis of ELISA data for healthy controls and patients suffering from different pathological stages of lung cancer (a) Exosomal GCC2 protein concentrations in healthy controls ($n = 16$) and patients with middle-stage (T2aN0-T2bN0) lung cancer ($n = 24$). (b) ROC curve analysis of the exosomal GCC2 protein concentrations in patients with middle-stage (T2aN0-T2bN0) lung cancer. (c) Exosomal GCC2 protein concentrations in healthy controls ($n = 16$) and patients with late-stage (T2aN1-T2bN1-T2aN2-T2bN2) lung cancer ($n = 16$). (d) ROC curve analysis of the exosomal GCC2 protein concentrations in patients with late-stage (T2aN1-T2bN1-T2aN2-T2bN2) lung cancer.

Table S1. (please find at excel file of Supplementary Table S1)

Table S2. Size of exosomes derived from the lung cancer cell lines and patient plasmas.

Group	Mean size (nm) ± S.D.	*p* value
Normal healthy Control (*n* = 16)	135.36 ± 5.31	0.36
Lung cancer Patients (*n* = 53)	138.71 ± 12.40	
**Cell line**	**Mean size (nm) ± S.D.**	***p* value**
HPAEpiC	134.131±1.99	
H1299	138.73±6.98	0.42
H522	131±3.2	0.27
